# Nucleotide Diversity of the Maize *ZmCNR13* Gene and Association With Ear Traits

**DOI:** 10.3389/fgene.2021.773597

**Published:** 2021-10-26

**Authors:** Zhihao Zuo, Yue Lu, Minyan Zhu, Rujia Chen, Enying Zhang, Derong Hao, Qianfeng Huang, Hanyao Wang, Yanze Su, Zhichao Wang, Yang Xu, Pengcheng Li, Chenwu Xu, Zefeng Yang

**Affiliations:** ^1^ Jiangsu Key Laboratory of Crop Genetics and Physiology Key Laboratory of Plant Functional Genomics of the Ministry of Education Jiangsu Key Laboratory of Crop Genomics and Molecular Breeding, Agricultural College of Yangzhou University, Yangzhou, China; ^2^ Jiangsu Co-Innovation Center for Modern Production Technology of Grain Crops, Yangzhou University, Yangzhou, China; ^3^ Joint International Research Laboratory of Agriculture and Agri-Product Safety of Ministry of Education of China, Yangzhou University, Yangzhou, China; ^4^ College of Agronomy, Qingdao Agricultural University, Qingdao, China; ^5^ Jiangsu Yanjiang Institute of Agricultural Sciences, Nantong, China

**Keywords:** maize, nucleotide polymorphism, ear-related traits, ZmCNR13, association analysis

## Abstract

The maize (*Zea mays* L*.*) *ZmCNR13* gene, encoding a protein of *fw2.2-like* (*FWL*) family, has been demonstrated to be involved in cell division, expansion, and differentiation. In the present study, the genomic sequences of the *ZmCNR13* locus were re-sequenced in 224 inbred lines, 56 landraces and 30 teosintes, and the nucleotide polymorphism and selection signature were estimated. A total of 501 variants, including 415 SNPs and 86 Indels, were detected. Among them, 51 SNPs and 4 Indels were located in the coding regions. Although neutrality tests revealed that this locus had escaped from artificial selection during the process of maize domestication, the population of inbred lines possesses lower nucleotide diversity and decay of linkage disequilibrium. To estimate the association between sequence variants of *ZmCNR13* and maize ear characteristics, a total of ten ear-related traits were obtained from the selected inbred lines. Four variants were found to be significantly associated with six ear-related traits. Among them, SNP2305, a non-synonymous mutation in exon 2, was found to be associated with ear weight, ear grain weight, ear diameter and ear row number, and explained 4.59, 4.61, 4.31, and 8.42% of the phenotypic variations, respectively. These results revealed that natural variations of *ZmCNR13* might be involved in ear development and can be used in genetic improvement of maize ear-related traits.

## Introduction

Maize (*Zea mays* L*.*), one of the most important cereal crops, is cultivated worldwide as sources of food, animal feed, and industrial materials. It was suggested that the total global maize production was 1,148.4 million tons in 2019, which was far greater than those of rice (*Oryza sativa* L*.*) and wheat (*Triticum aestivum* L*.*)^1^. However, improving kernel yield (KY) is still a primary mission in maize breeding (Li et al., 2018). KY is a complex quantitative trait affected by a variety of genetic and environmental factors and has low heritability (Beavis et al., 1994; Yan et al., 2006). Compared with KY, the heritability of yield components is relatively higher and is less affected by environmental factors (Messmer et al., 2009; Li et al., 2013; Raihan et al., 2016). Therefore, it is more effective to select some yield components than to directly select KY in the breeding process (Sidwell et al., 1976). Among the yield components, kernel size has a crucial effect on kernel weight estimated by kernel length (KL), kernel width (KW), and kernel thickness (KT). In addition, ear length (EL), ear diameter (ED), ear row number (ERN), and kernel number per row (KNR) are essential traits determining the kernel number (Zhang et al., 2017). All of these ear-related traits are quantitative traits regulated by multiple genes and environmental factors (Liu et al., 2012). Many genes regulating maize ear-related traits have been identified, such as *fea2* (Bommert et al., 2013), *fea3* (Je et al., 2016), *KNR1* (Wang et al., 2019) and *KNR4* (Liu et al., 2015) for kernel row number, *td1* (Bommert et al., 2005), *bif2* (McSteen and Hake 2001) and *ba1* (Ritter et al., 2002) for tassel morphology.

Tomato *fruit-weight 2.2* (*fw2.2*) was detected as an essential locus in controlling fruit weight and size (Frary et al., 2000; Nesbitt and Tanksley 2001). In plants, the homologs of *fw2.2-like (FWL)* genes, encoding proteins with a conserved PLAC8 (named after a series of human placental specific protein with unknown function) domain, were suggested to play essential roles in cell division and organ size control (Libault and Stacey 2010). A total of eight members of *FWL* gene family in rice were detected. Among them, *OsFWL1* and *OsFWL3* genes modulate rice grain length by regulating cell number, and *OsFWL4* gene is a negative regulator of tiller number and plant yield (Xu et al., 2013; Gao et al., 2020). In soybean, the silencing of *GmFWL1* expression resulted in a significant decrease in the number of nodules and changes in the structure of cell chromatin (Libault et al., 2010). In maize, a total of 13 *FWL* gene family members were identified, and named as *Cell Number Regulator (CNR)* genes (Guo et al., 2010). The *ZmCNR1* gene was illustrated to possess a plant-specific cell proliferation function affecting plant and fruit weight. In addition, it was also suggested that the expression of *ZmCNR2* negatively correlate with tissue growth activity and hybrid seedling vigor (Guo et al., 2010). These observations revealed that plant *FWL/CNR* genes play critical roles in plant development.

The maize *CNR* gene *ZmCNR13* was firstly identified through a *narrow odd dwarf* (*nod*) mutant (Rosa et al., 2017). Further evidence revealed that the *ZmCNR13* gene possessed the function in regulating cell division and differentiation and then affected both vegetative and reproductive development of maize (Rosa et al., 2017). However, the effect of this gene in the regulation of maize ear-related traits remains largely unknown, and there is no association analysis between the nucleotide polymorphisms of the maize *ZmCNR13* gene and yield-related traits. In this study, we investigated the nucleotide polymorphism of the maize *ZmCNR13* locus, and estimated the association between the sequence polymorphisms of this gene and some ear-related traits.

## Materials and Methods

### Plant Materials, Experimental Design, and Analysis of Phenotypic Data

A total of 224 inbred lines (Supplementary Table S1) have been selected for phenotypic observation in this study. These lines had been grown in the field in a randomized block design with two replicates at Sanya (18°23′ N, 109°44′ E) in 2015, 2016 and Yangzhou (32°39′ N, 119°42′ E) in 2017. Each line was planted in a sequential row patterns with 15 plants, 3.5 m long and 0.4 m between adjacent rows. After harvesting and drying, three well-developed ears have been selected to measure ear traits, including ear weight (EW), ear grain weight (EGW), EL, ED, ERN, KNR, hundred kernel weight (HKW), KL, KW, and KT. ANOVA was performed using “aov” function in R software. The “lme4” (Bates et al., 2015) package was used to calculated the broad-sense heritability (*h* ^*2*^) for these ear-related traits. The observed values of these traits in three environments were used to calculate the best linear estimated prediction (BLUP) (Piepho et al., 2008) using the package ‘lme4’. The calculation model of BLUP is: $y_{ij}=\mu +f_{j}+e_{i}+\epsilon_{ij}$ , where *y*_*ij*_ is the observed value of the phenotype of *j* line in *i* environment, *μ* is the phenotypic mean of *j* line in all environments, *f*_*j*_ is the genetic effect of *j* line, *e*_*i*_ is the environment effect of *i* and *ε*_*ij*_ is the error of the observed value of the *j* line in *i* environment. The descriptive statistics and correlation coefficients were estimated using the “GGally” package in R software.

### DNA Extraction and *ZmCNR13* Re-Sequencing

A total of 224 inbred lines, 56 landraces and 30 teosintes (Supplementary Table S1) were collected for target capture sequencing. Fresh and young leaves were collected from each line at the seeding stage, and a modified cetyl trimethyl ammonium bromide (CTAB) method was used to extract genomic DNA (Allen et al., 2006). DNA sequences of the *ZmCNR13* locus in the selected lines were resequenced using the target sequence capture sequencing technology on the NimbleGen platform (Choi et al., 2009) by BGI (Beijing Genomics Institute) Life Tech Co. The genomic sequences and positions of the *ZmCNR13* (GRMZM2G027821) locus in the inbred line B73 (AGPv3.21) were used as the references for target capture sequencing.

### Analysis of Genotypic Data

The software Clustal X (Larkin et al., 2007) was used for multiple sequence alignment of the *ZmCNR13*. The nucleotide polymorphisms and allelic diversities of all tested lines were analyzed by DNASP5.0 software (Librado and Rozas 2009). Nucleotide diversity (*π*) in the *ZmCNR13* gene, which is defined as the mean number of nucleotide differences per site between any two DNA sequences, was estimated using R package “PopGenome” (Pfeifer et al., 2014). Linkage disequilibrium (LD) decay was measured by the squares of correlation coefficients (*R* ^*2*^) for all pairs of SNPs using the program PopLDdecay v3.41 (Zhang et al., 2019) with default parameters.

### Marker-Trait Association Analysis in Inbred Lines

The method of genotyping-by-sequencing (GBS) was used to identify the genotypes of the selected lines (Li et al., 2019a). A total of 361,675 SNPs, which were distributed across the entire maize genome, were used to calculate the population structure and kinship. The population structure was estimated through the method of principal components (PCs). In this analysis, the top five PCs, which can explain 23.56% genetic variation, were used for association mapping. In addition, pair-wise coefficients of relatedness (kinship matrix) was calculated by TASSEL5.0 (Bradbury et al., 2007). In order to increase the accuracy of association analysis, two models were used for marker-trait association analysis. The general linear model (GLM) (Pritchard et al., 2000; Zhao et al., 2007) controlling for population structure (PCs), and mixed linear model (MLM) (Yu et al., 2006) controlling for both population structure (PCs) and kinship, were calculated by TASSEL5.0. A total of 398 markers in *ZmCNR13* with minor allele frequency (MAF) higher than 0.05 were used for association analysis. The *p*-value thresholds were empirically set to 1/398 and 0.05/398 for MLM and GLM, respectively, using the Bonferroni correction method (Bland and Altman 1995).

## Results

### The Phenotypic Variations Among Maize Inbred Lines

**FIGURE 1:**
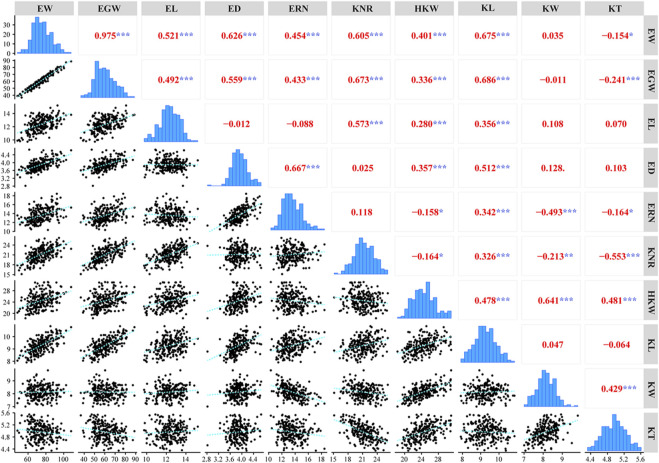
Phenotypic distribution and Pearson correlation coefficients for ten ear-related traits (***, *p* < 0.001; **, *p* < 0.01; *, *p* < 0.05).

In this study, a total of ten ear-related traits, including EW, EGW, EL, ED, ERN, KNR, HKW, KL, KW, and KT, were obtained in a population of 224 maize inbred lines (Table 1). Coefficients of variation of these traits ranged from 12.11 to 40.79%, suggesting abundant phenotypic diversity among the tested inbred lines. ANOVA analyses also revealed that all these ear-related traits showed significant difference among inbred lines, suggesting that this population hold genetic characteristics for association analysis. In addition, we also noticed that both the environments and genotype-environment interaction had a significant impact on all these traits. The values of broad-sense heritability were further estimated. The results revealed that most of these traits possessed high heritability. The estimated heritability for ED, ERN, HKW, KL, and KW is higher than 40%. To evaluate the correlation relationship among these ten ear-related traits, paired correlation analysis was carried out, and the estimations of correlation coefficient (*r*) between any two traits were obtained (Figure 1). Significant correlations were observed between most parameters. Among them, EW/EGW had the highest correlation with *r* = 0.975. Meanwhile, the 12 of 45 pairwise correlations for ear-related traits, including ED/EL, ERN/EL, KNR/ED, KNR/ERN, KW/EW, KW/EGW, KW/EL, KW/ED, KW/KL, KT/EL, KT/ED and KT/KL didn’t reach the significant level. These results indicate that different genetic mechanisms might affect the ear traits of maize.

**TABLE 1:** Descriptive statistics and ANOVA results of the maize ear-related traits.

Trait	Mean	*SD*	Min	Max	*CV*	*F* Value (G)	*F* Value (E)	*F* Value (G×E)	*h* ^*2*^ (%)
EW (g)	77.04	29.10	6.02	214.10	37.77	13.52^a^	178.37^a^	4.15^a^	28.04
EGW (g)	63.09	25.73	3.66	184.00	40.79	13.72^a^	128.18^a^	4.27^a^	28.03
EL (cm)	12.14	2.44	3.77	21.54	20.14	17.30^a^	545.33^a^	3.67^a^	38.08
ED (cm)	3.91	0.50	1.01	8.63	12.75	16.87^a^	113.09^a^	2.79^a^	47.15
ERN	13.58	2.40	6.00	22.00	17.65	21.57^a^	71.86^a^	2.24^a^	52.97
KNR	21.06	5.88	1.00	42.00	27.93	11.65^a^	128.32^a^	3.26^a^	28.56
HKW (g)	25.58	5.95	10.20	44.44	23.25	35.97^a^	2223.19^a^	7.92^a^	58.30
KL (mm)	9.46	1.39	4.93	33.73	14.72	9.78^a^	101.88^a^	2.02^a^	41.50
KW (mm)	8.12	0.98	5.22	25.20	12.11	10.44^a^	37.11^a^	1.45^a^	45.80
KT (mm)	5.08	0.91	2.58	15.23	17.82	8.71^a^	610.32^a^	2.91^a^	22.65

a Indicates statistical significance at p < 0.001 level.

Abbreviationsin the table are as follows: CV, coefficient of variation,; G, genotype; E, environment; G×E, genotype-environment interaction; h^2^, broad-sense heritability; EW, ear weight; EGW, ear grain weight; EL ear length; ED, ear diameter; ERN, ear row number; KNR, kernel number per row; HKW, hundred kernel weight; KL, kernel length; KW, kernel width and KT, kernel thickness.

### Sequence Polymorphisms of the Maize *ZmCNR13* Gene

**FIGURE 2:**
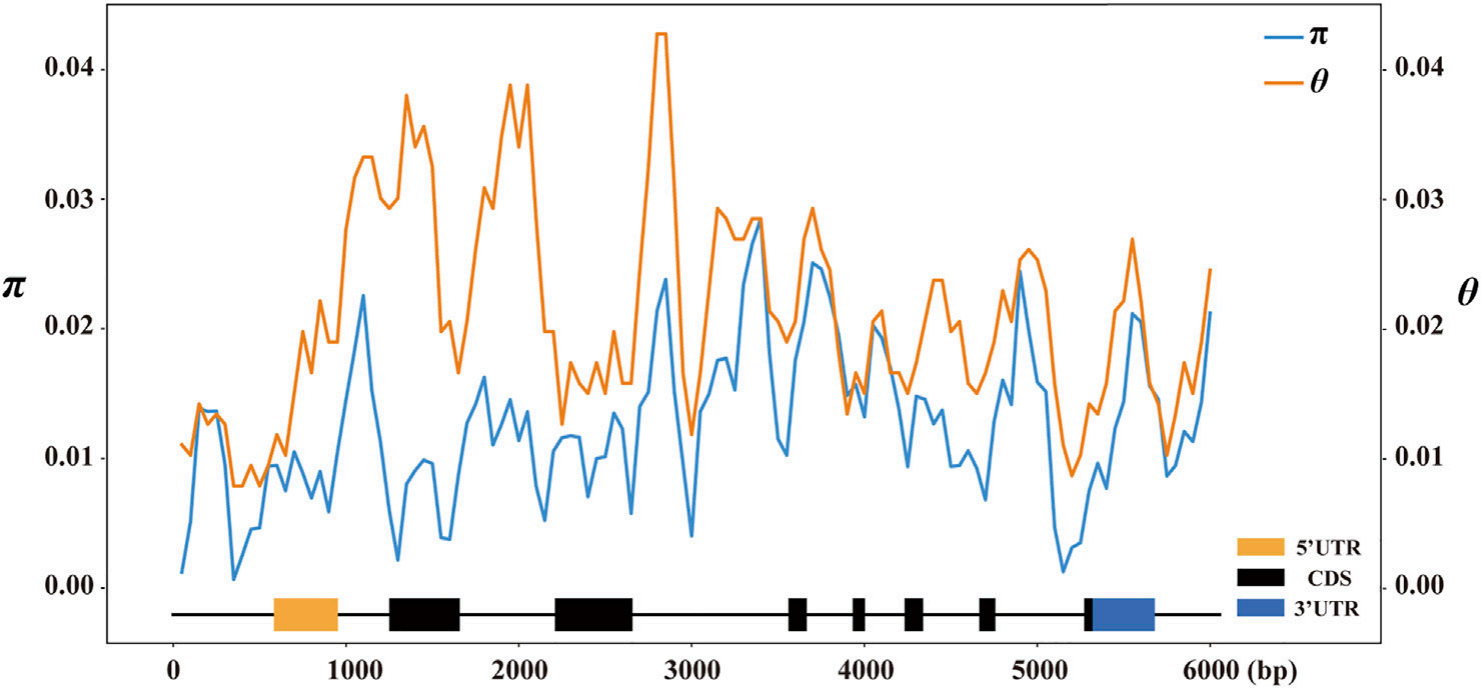
Nucleotide diversity (*π* and *θ*) estimated along the sequences of maize *ZmCNR13. π* and *θ* were calculated using the method of sliding windows of 200 bp with a step of 50 bp.

To evaluate the sequence polymorphisms of the *ZmCNR13* gene, the full-length sequences of this locus were re-sequenced in 224 inbred lines. After multiple sequence alignment, a full-length 6,197 bp sequence was obtained, including 582 bp upstream region, 5,098 bp coding region containing seven exons and seven introns, and 517 bp downstream region. A total of 501 variants were identified in the genomic sequence, including 415 SNPs and 86 indels covering 306 sites. On average, SNPs and Indels occurred per 12.37 bp and 72.06 bp, respectively. The highest frequency of variation sites was detected in the intron region (11.28 bp for SNP and 58.11 bp for InDels). The frequency of variation sites in the exon region was the lowest (23.23 bp for SNP and 321.75 bp for InDels). For all the tested lines, the overall nucleotide diversity (*π*) of the *ZmCNR13* locus was 0.01719, where the estimated *π* values of intron regions were relatively higher than exon regions. The nucleic acid polymorphisms of exon regions were low (*θ* = 0.00765), while that of other regions were relatively higher (*θ* = 0.01702 for intron regions and 0.01544 for downstream) (Table 2). In addition, *π* and *θ* were calculated using the sliding window of 200 bp with a step length of 50 bp. Two peaks in the intron3 and intron7 revealed that these regions were more diverse than others (Figure 2).

**TABLE 2:** Summary of parameters for the analysis of nucleotide polymorphisms.

Parameters	Upstream	5′UTR	Exons	Introns	3′UTR	Downstream	Full-length
Total length of amplicons (bp)	582	371	1,287	3,080	360	517	6,197
Number of all of the sequence variants	30	17	55	336	26	50	501
Frequency of all of the sequence variants	0.0515	0.0458	0.0427	0.1091	0.0722	0.0967	0.0808
Number of polymorphic sites	21	13	51	273	21	37	415
Frequency of polymorphic sites per bp	0.0361	0.0350	0.0396	0.0886	0.0583	0.0716	0.0670
Number of indels sites	31	11	60	120	37	48	306
Number of indels events	9	4	4	53	5	13	86
Average Indel length	3.4444	2.75	15	2.2642	7.4	3.6923	3.5581
Frequency of indels per bp	0.0155	0.0108	0.0031	0.0172	0.0139	0.0251	0.0139
*π*	0.00902	0.01093	0.00971	0.02090	0.02254	0.02391	0.01719
*θ*	0.00816	0.00613	0.00765	0.01702	0.01132	0.01544	0.01312
Tajima’s *D*	0.2807	1.9086	0.7962	0.7203	2.6444^a^	1.5757	0.9854
Fu and Li’s *D*	−1.2487	1.4749	1.0715	1.2195	1.2724	0.4676	1.1647
Fu and Li’s *F*	−0.7670	1.9666^a^	1.1408	1.1546	2.1924^b^	1.1370	1.2750

a Indicates a statistical significance at p < 0.05 level.

b Indicates a statistical significance at p < 0.01 level. “UTR” indicates untranslated region.

### Nucleotide Diversity and Selection of *ZmCNR13* in Inbred Lines, Landraces and Teosintes

**FIGURE 3:**
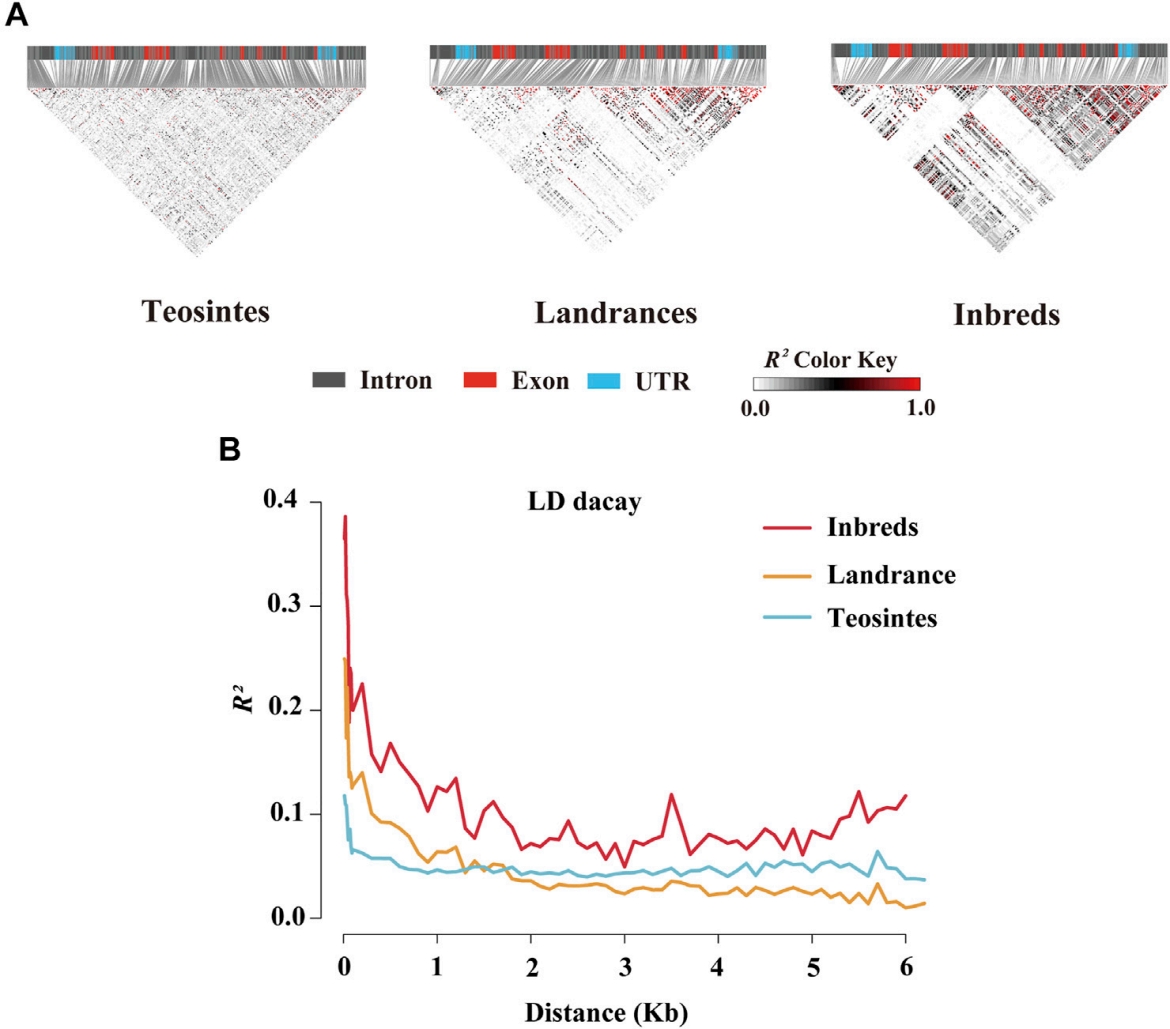
Linkage disequilibrium (LD) analysis of *ZmCNR13* gene in three populations. **(A)** LD model of *ZmCNR13* gene in three populations. **(B)** LD decay for three populations measured by *R* ^*2*^ *.*

To further estimate the genetic diversity of the *ZmCNR13* locus, the sequence variation parameters of this gene in three different populations were analyzed and compared. The sequence conservation (C) of the *ZmCNR13* gene was similar in landraces (C = 0.903) and inbred lines (C = 0.914), and the lowest value of 0.813 in teosintes. Correspondingly, compared with teosintes, landraces and inbred lines showed lower nucleotide sequence polymorphisms (*θ* = 42.25 for teosintes, 19.01 for landraces and 13.12 for inbred lines). The neutrality of *ZmCNR13* gene was tested by Tajima’s *D*, Fu and Li’s D*, and Fu and Li’s F*. The Tajima’s *D* values of the three populations didn’t achieve a significant level. Furthermore, the Tajima’s *D* was positive in the inbred line population, indicating that the gene was lack of rare alleles in this population (Table 3 and Supplementary Figure S1). There is no prominent LD block of the *ZmCNR13* gene in teosintes, and the degree of LD between mutation sites is relatively low. Compared with teosintes, the LD degree of landraces is enhanced to a certain extent, and smaller blocks begin to appear. In inbred lines, further the degree of LD is enhanced and larger blocks appear (Figure 3A). We also estimated the attenuation of LD with physical distance in three populations. The result revealed that the LD attenuation rate of inbred lines was slower than that of landraces and teosintes, and *R* ^*2*^ decreased to less than 0.2 after 200 bp, while those of landraces and teosintes decreased to less than 0.2 when it was less than 100 bp (Figure 3B). Taken together, although the gene didn’t escape from neutral evolution, bottleneck effect of population has led to the lower nucleotide polymorphisms and LD decay in inbred lines.

**TABLE 3:** Summary of nucleotide polymorphisms and neutrality test of *ZmCNR13*.

Populations	C	π × 1,000	*θ* × 1,000	Tajima’s *D*	Fu and Li’s *D*	Fu and Li’s *F*
teosintes	0.813	31.94	42.25	−0.9542	−1.3684	−1.4553
landraces	0.903	17.02	19.01	−0.3774	−0.5176	−0.5548
inbreds	0.914	17.19	13.12	0.9854	1.1647	1.2750
all	0.777	18.61	31.00	−1.2499	−4.5758^b^	−3.2829^b^

a Indicates a statistical significance at p < 0.05 level.

b Indicates a statistical significance at p < 0.01 level.

### Association Analysis of Ear-Related Traits With *ZmCNR13*

**FIGURE 4:**
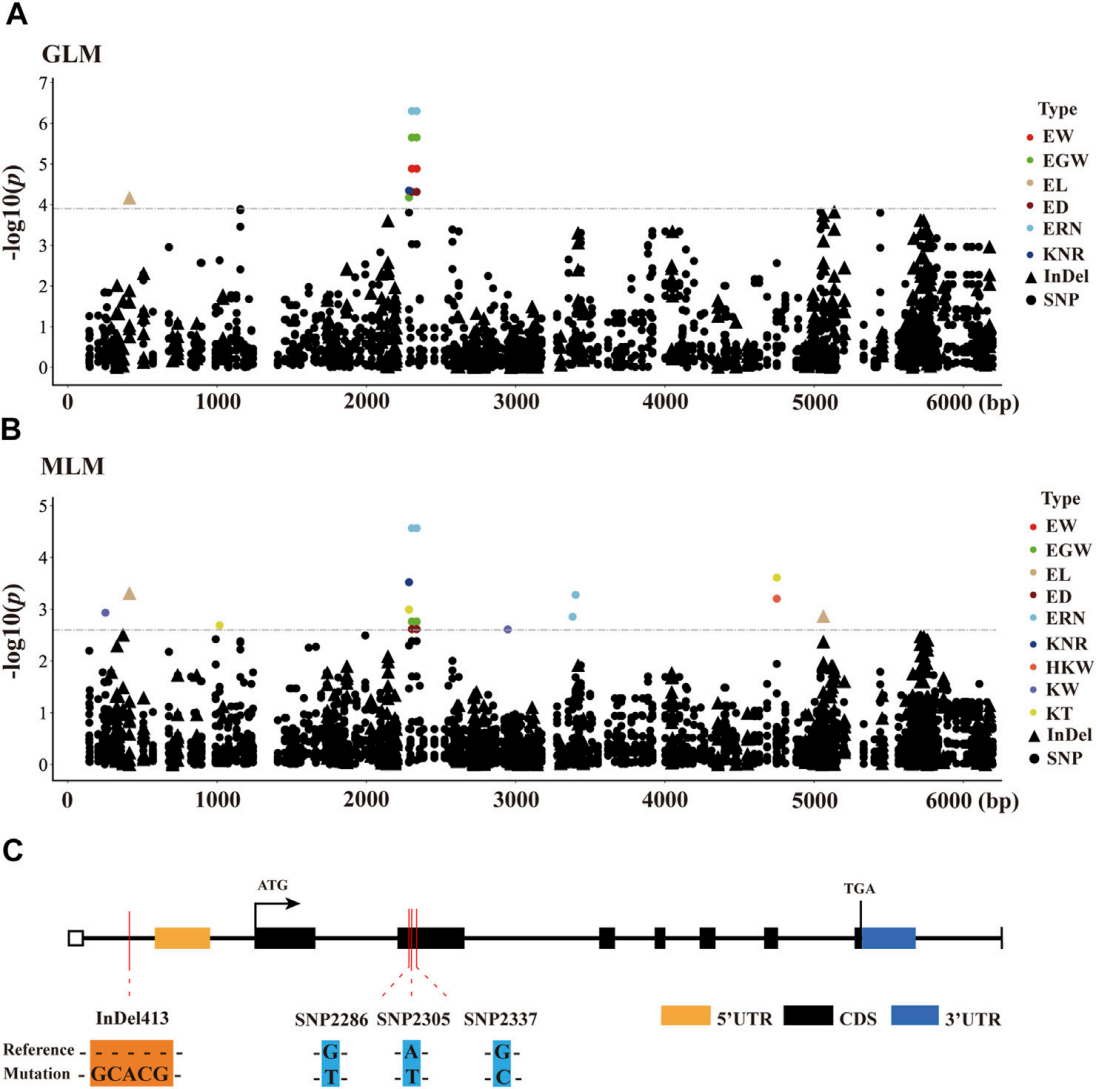
Candidate-gene based association of *ZmCNR13* gene in inbred lines. **(A,B)** Manhattan plot using the MLM and GLM model. The *p*-value threshold was set at 1/398 (MLM) and 0.05/398 (GLM). Triangles and dots represent InDels and SNPs, respectively. **(C)** Schematic of the *ZmCNR13* gene structure and allelic variation.

To identify significant variants associated with ear-related traits, a total of 398 markers in *ZmCNR13* with minor allele frequency (MAF) higher than 0.05 were used for association analysis. Both methods of GLM and MLM were used for marker-trait association analysis. Four polymorphic sites (InDel413, SNP2286, SNP2305 and SNP2337) were captured jointly by two models, which were found to be significantly associated with six ear traits (EW, EGW, EL, ED, ERN, and KNR). Among them, two sites (SNP2305 and SNP2337) located in exon 2 jointly controlled four ear traits (EW, EGW ED and ERN). Furthermore, we noticed that SNP2305 belongs to non-synonymous mutation, where the transition of T to A will lead to the changes of amino acid serine to threonine. In addition, SNP2305 were found to be associated with EW, EGW, ED and ERN, and could explain 4.31–8.42% and 7.06–10.41% phenotypic variations under MLM and GLM, respectively (Figure 4 and Supplementary Table S2).

**FIGURE 5:**
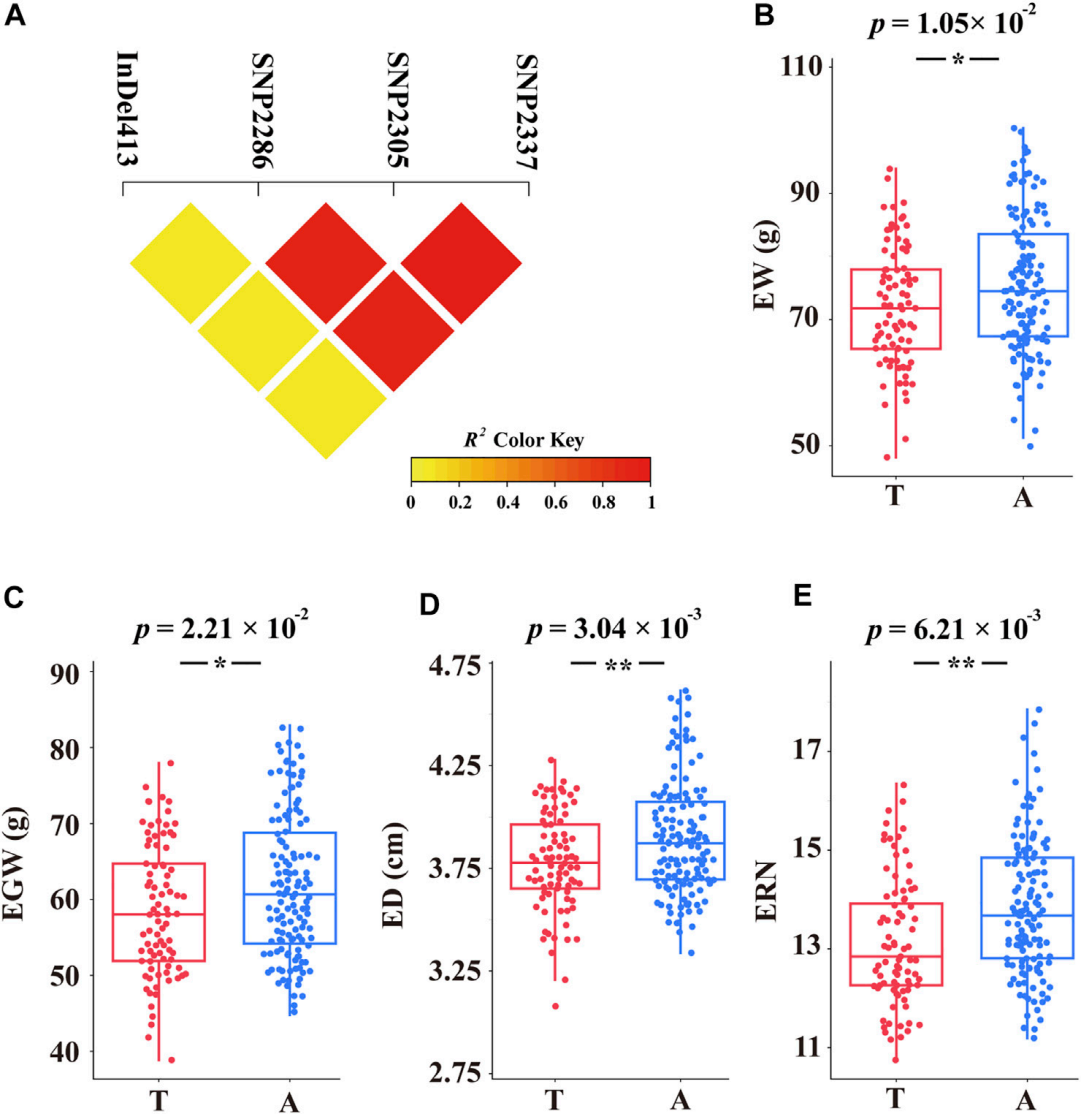
Natural variations in *ZmCNR13* were significantly associated with four ear-related traits. **(A)** LD heatmap for four significant variants associated with ear traits. **(B–E)** Comparison of EW, EGW, ED and ERN between different alleles of SNP2305. *p* value for *t* test comparing two groups carrying different alleles were indexed on the top (**, *p* < 0.01; *, *p* < 0.05).

LD analysis showed that SNP2286, SNP2305 and SNP2337 had relatively high linkage across inbred lines. Interestingly, SNP2305 and SNP2337 had complete linkage (*R* ^*2*^ = 1) (Figure 5A). We further classified haplotypes based on the variation of SNP2305, and divided the inbred lines into two major groups. *T*-test revealed that EW, EGW, ED and ERN showed significant difference between two groups. The allele A group possessed significantly higher values of EW, EGW, ED and ERN than the allele T (Figures 5B–E). On this basis, combined with correlation analysis, we noticed that the changes among the four traits were synergistic. Therefore, we can infer that allele A of SNP2305 has positive regulatory effects on EW, EGW, ED and ERN.

## Discussion

In the present study, association analyses were employed to illustrate the relationship between the maize *ZmCNR13* and ear-related traits. Association analysis, also known as linkage disequilibrium mapping or association mapping, is a method based on linkage disequilibrium to identify the association between genetic polymorphisms and phenotypic variations (Mackay and Powell 2007). Compared with linkage analysis based on parental population, association analysis has higher resolution, and it can be directly used to analyze more than two alleles in natural populations (Salvi and Tuberosa 2005; Mackay and Powell 2007). As an extension of genome-wide association mapping, candidate gene association analysis is mainly used to identify genetic variations and excellent haplotypes significantly associated with target traits (Yan et al., 2011). Maize is a typical cross-pollinated plant with a high recombination rate, rich genetic diversity and LD decay distance of about 1Kb, so it is an ideal material for association analysis (Tenaillon et al., 2001; Whitt et al., 2002). Many loci affecting maize traits have been identified by this method, such as *Zmisa2* (Yang et al., 2014) and *ZmBT1* (Xu et al., 2014) for starch properties, *ZmYS1* (Yang et al., 2015) for kernel mineral concentrations, *ZmCKX5* (Wang et al., 2021) and *ZmMADS60* (Li et al., 2020) for root morphology, *ZmHKT1* (Li et al., 2019a) and *ZmPGP1* (Li et al., 2019b) for plant architecture.

Abundant genetic diversity is the basis for crop improvement (Yan et al., 2011). In this study, nucleotide polymorphisms of the *ZmCNR13* gene were analyzed in inbred lines through re-sequencing. A total of 501 variations including 415 SNPs and 86 InDels were detected, and most of them concentrated in the intron regions. The exon region had one SNP per 25.71 bp, while the intron region reached one SNP per 11.28 bp. The decrease of nucleotide polymorphism in exon region suggested that these regions might be influenced by greater selection pressure. Moreover, it is worth noted that the LD decays faster in landraces and teosintes than inbred lines, suggesting that there were genetic bottleneck effects (Wang et al., 1998) in the population of inbred lines, although no obvious selection signature was detected through neutral test.

Ear-related traits are the most important components of kernel yield of maize. Illustrating the genetic background of the genes related to ear traits and digging their elite variations will be of great importance in high-yield breeding maize (Zhu et al., 2018). In the present study, we revealed that a non-synonymous mutation in exon 2 (SNP2305) of the *ZmCNR13* gene was found to be significantly associated with four ear-related traits, including EW, EGW, ED and ERN. In addition, we further noticed that the inbred lines carrying SNP2305A possess higher EW, EGW, ED and ERN than those carrying SNP2305T. The validity of candidate gene association analysis has been repeatedly confirmed (Yan et al., 2011). For instance, the association between polymorphisms of *dwarf8* and flowering time was detected by some independent works (Thornsberry et al., 2001; Andersen et al., 2005; Camus-Kulandaivelu et al., 2006). To confirm the results of association analysis, we compared the phenotypes between the inbred lines carrying SNP2305A and SNP2305T in three environments. The results revealed that there are statistical differences between them for all these traits in different environments (Supplementary Figure S2), suggestive the genetical credibility of the association results. These observations revealed that the superior allelic variations of *ZmCNR13* possess potential application values in maize genetic improvement.

In conclusion, the maize *ZmCNR13* gene was re-sequenced in 224 inbred lines, 56 landraces and 30 teosintes. Although this gene was escaped from artificial selection during maize genetic improvement, a non-synonymous mutation in exon2 was found to be associated with ear-related traits, including EW, EGW, ED and ERN. The superior allelic variations of *ZmCNR13* has potential application values in maize genetic improvement.

## Data Availability

The original contributions presented in the study are publicly available. This data can be found here:PRJNA764471.

## Footnotes

1
http://www.fao.org/faostat/zh/#data/QCL.2019
