# Supplementary material for: Nucleotide Diversity of the Maize ZmCNR13 Gene and Association With Ear Traits
Source: Front Genet. 2021 Oct 26;12:773597. doi: 10.3389/fgene.2021.773597 (PMC8576287; doi:10.3389/fgene.2021.773597)
Supplement: Supplementary file 1 [file DataSheet2.PDF]

Supplementary Table S2. Association analysis results using the MLM and GLM models.

Trait	Marker	Alleles	Region	MLM			GLM		
				p Value	-lg(*p*)	R^2	*p* Value	-lg(*p*)	R^2
EW	SNP2305	A/T	exon 2	1.74×10^{-3}	2.7595	0.0459	1.31×10^{-5}	4.88	0.0821
EW	SNP2337	G/C	exon 2	1.74×10^{-3}	2.7595	0.0459	1.31×10^{-5}	4.88	0.0821
EGW	SNP2286	G/T	exon 2	/	/	/	6.68×10^{-5}	4.18	0.0693
EGW	SNP2305	A/T	exon 2	1.72×10^{-3}	2.7645	0.0461	2.24×10^{-6}	5.65	0.0961
EGW	SNP2337	G/C	exon 2	1.72×10^{-3}	2.7645	0.0461	2.24×10^{-6}	5.65	0.0961
EL	InDel413	-----/GCACG	upstream	4.91×10^{-4}	3.3087	0.0561	6.84×10^{-5}	4.16	0.0607
EL	SNP5062	A/-	intron 7	1.37×10^{-3}	2.8633	0.0472	/	/	/
ED	SNP2305	A/T	exon 2	2.43×10^{-3}	2.6144	0.0431	4.85×10^{-5}	4.31	0.0706
ED	SNP2337	G/C	exon 2	2.43×10^{-3}	2.6144	0.0431	4.85×10^{-5}	4.31	0.0706
ERN	SNP2305	A/T	exon 2	2.72×10^{-5}	4.5646	0.0842	5.01×10^{-7}	6.30	0.1041
ERN	SNP2337	G/C	exon 2	2.72×10^{-5}	4.5646	0.0842	5.01×10^{-7}	6.30	0.1041
ERN	SNP3382	C/T	intron 3	1.40×10^{-3}	2.8539	0.0480	/	/	/
ERN	SNP3402	G/A	intron 3	5.30×10^{-4}	3.2756	0.0567	/	/	/
KNR	SNP2286	G/T	exon 2	3.02×10^{-4}	3.5202	0.0617	4.51×10^{-5}	4.35	0.0725
HKW	SNP4750	G/A	exon 6	6.27×10^{-4}	3.2031	0.0507	/	/	/
KW	SNP252	G/C	upstream	1.17×10^{-3}	2.9318	0.0486	/	/	/
KW	SNP2948	A/G	intron 3	2.46×10^{-3}	2.6091	0.0421	/	/	/
KT	SNP1017	T/C	intron 1	2.05×10^{-3}	2.6882	0.0416	/	/	/
KT	SNP2286	G/T	exon 2	1.02×10^{-3}	2.9914	0.0474	/	/	/
KT	SNP4750	G/A	exon 6	2.46×10^{-4}	3.6083	0.0594	/	/	/
