# Supplementary figures and images for: Nucleotide Diversity of the Maize ZmCNR13 Gene and Association With Ear Traits
Source: Front Genet. 2021 Oct 26;12:773597. doi: 10.3389/fgene.2021.773597 (PMC8576287; doi:10.3389/fgene.2021.773597)

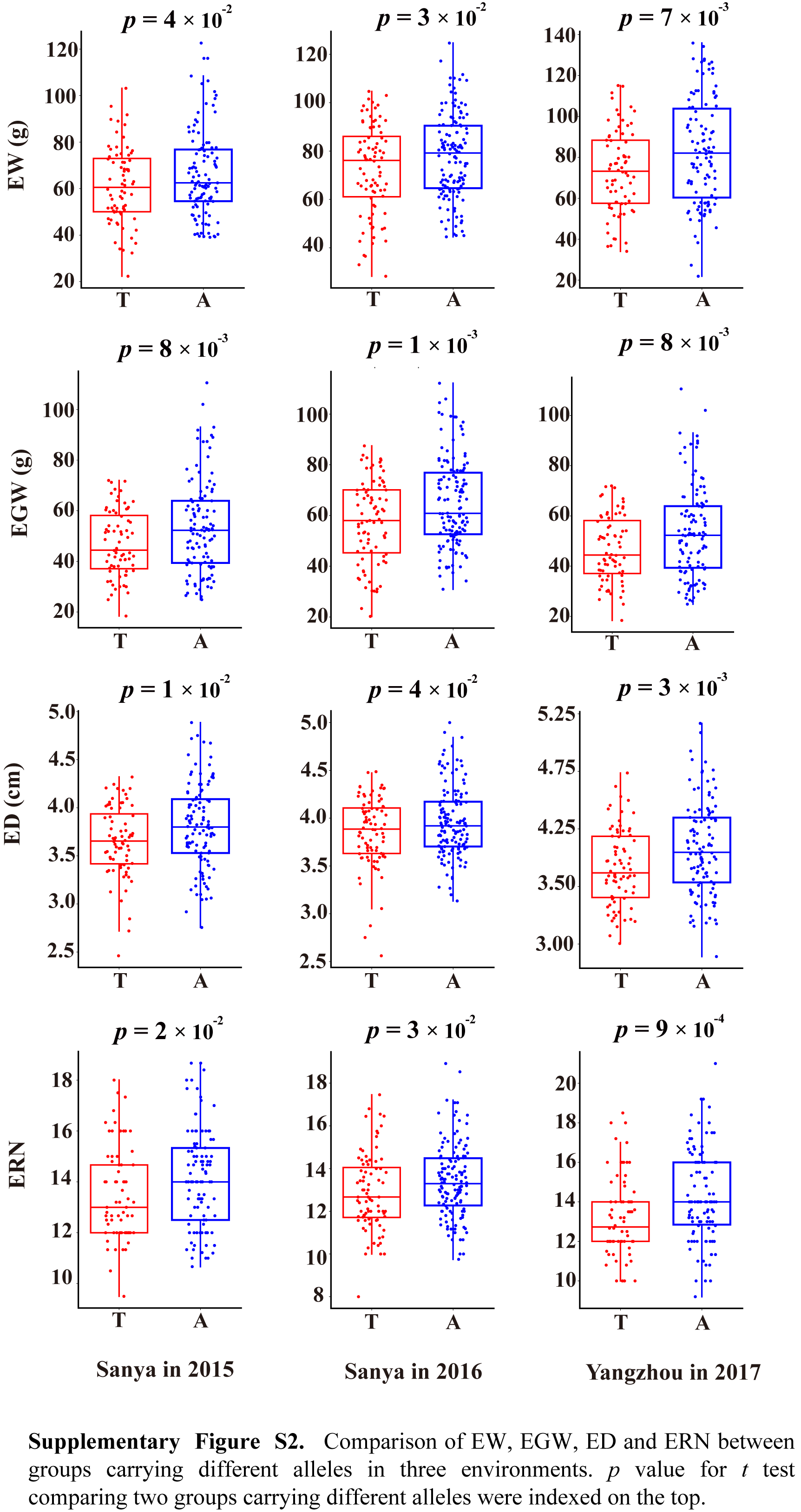

Supplement: Supplementary file 2 [file Image2.TIF]

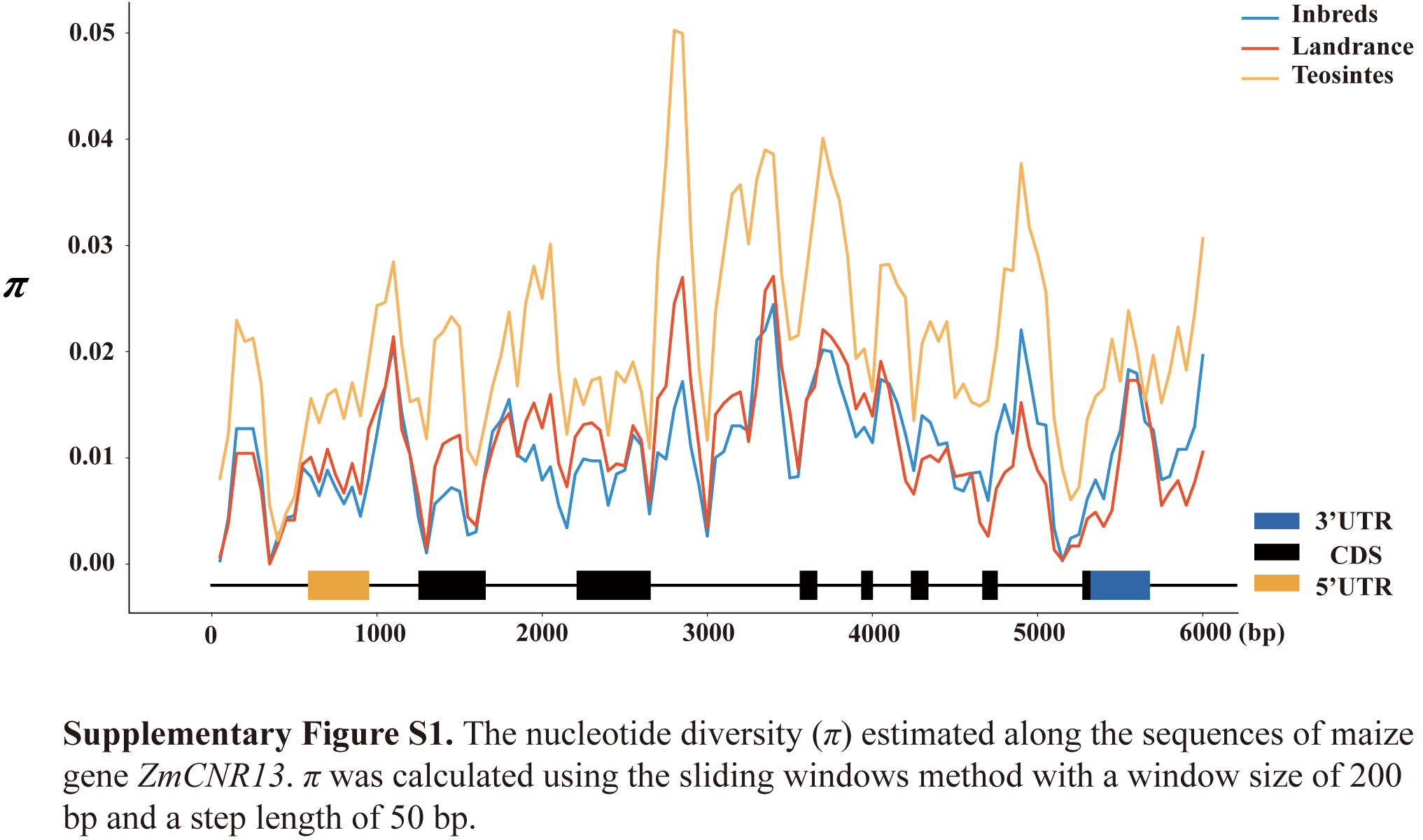

Supplement: Supplementary file 3 [file Image1.TIF]
