# Supplementary material for: Nucleotide Diversity of the Maize ZmCNR13 Gene and Association With Ear Traits
Source: Front Genet. 2021 Oct 26;12:773597. doi: 10.3389/fgene.2021.773597 (PMC8576287; doi:10.3389/fgene.2021.773597)
Supplement: Supplementary file 4 [file DataSheet1.PDF]

Supplementary Table S1. The list of 224 inbred lines, 56 landraces and 30 teosintes used in this study.

Category	Line ID	Line Name	Group
Inbred line	A004	107	Tang SPT
Inbred line	A005	Huang518	Tang SPT
Inbred line	A006	K12	Tang SPT
Inbred line	A007	H21	Tang SPT
Inbred line	A008	Ji853	Tang SPT
Inbred line	A010	Huangzaosi	Tang SPT
Inbred line	A011	502	Tang SPT
Inbred line	A012	Luyuan92	Tang SPT
Inbred line	A013	10168	Tang SPT
Inbred line	A014	QZ01	Tang SPT
Inbred line	A015	Y53	Tang SPT
Inbred line	A017	Zong3-1	Lvdahonggu
Inbred line	A020	Zi330	Reid
Inbred line	A021	S122	Lvdahonggu
Inbred line	A024	Dan99	Lvdahonggu
Inbred line	A025	Nx335	Lancaster
Inbred line	A027	4CV	Lancaster
Inbred line	A029	OH43	Lancaster
Inbred line	A030	MO17	Lancaster
Inbred line	A031	BJ-4	Lancaster
Inbred line	A032	BEM	Lancaster
Inbred line	A033	BJ-5	Lancaster
Inbred line	A034	8112	Reid
Inbred line	A035	K8112	Reid
Inbred line	A037	4866	Reid
Inbred line	A038	3189	Reid
Inbred line	A039	Tie9206	Reid
Inbred line	A040	Benyu15	Reid
Inbred line	A041	Chun2433	Reid
Inbred line	A042	478s	Reid
Inbred line	A043	Zheng58	Reid
Inbred line	A044	7922	Reid
Inbred line	A045	5-Feb	Reid
Inbred line	A046	JB	Reid
Inbred line	A047	S80	Pgroup
Inbred line	A049	178	Pgroup
Inbred line	A052	Xy35	Pgroup
Inbred line	A054	6819	Pgroup
Inbred line	A055	Dan988	Pgroup
Inbred line	A056	319B	Pgroup
Inbred line	A057	Qi319w	Pgroup
Inbred line	A058	Qi318	Pgroup
Inbred line	A059	Shen137	Pgroup
Inbred line	A060	11099	Tropic
Inbred line	A062	11118	Tropic
Inbred line	A063	11200	Tropic
Inbred line	A064	10533-1	Tropic
Inbred line	A065	RCML15	Tropic
Inbred line	A066	RBS11	Tropic
Inbred line	A067	FLB01	Tropic
Inbred line	A069	Q52	Other
Inbred line	A071	QF-11	Other

Inbred line	A072	QF01	Other
Inbred line	A073	JND-1	Other
Inbred line	A075	BJ-2	Lancaster
Inbred line	A076	QF02	Other
Inbred line	A077	QF03	Other
Inbred line	A079	M1	Other
Inbred line	A081	WT26	Other
Inbred line	A082	M1132	Other
Inbred line	A097	Oh43-1	Lancaster
Inbred line	A098	Oh43-2	Lancaster
Inbred line	A099	Mo113	Lancaster
Inbred line	A102	S187	Lancaster
Inbred line	A103	LH39	Lancaster
Inbred line	A104	Mo17.351	Lancaster
Inbred line	A105	Mo17.352	Lancaster
Inbred line	A106	LH53	Lancaster
Inbred line	A113	13N-207	Pgroup
Inbred line	A114	13N-209	Pgroup
Inbred line	A115	Nongda1145	Pgroup
Inbred line	A116	T249	Pgroup
Inbred line	A117	YJ7	Pgroup
Inbred line	A118	S651	Pgroup
Inbred line	A119	CM	Pgroup
Inbred line	A120	Qi319X7	Pgroup
Inbred line	A121	JS06730	Pgroup
Inbred line	A122	Zong3.T877	Pgroup
Inbred line	A123	JS045	Pgroup
Inbred line	A125	Xin19M	Reid
Inbred line	A126	K22	Reid
Inbred line	A128	478	Reid
Inbred line	A131	H991	Reid
Inbred line	A134	Xian3M	Reid
Inbred line	A141	Zong31s	Lvdahonggu
Inbred line	A142	Z25F	Lvdahonggu
Inbred line	A144	13N-282	Lvdahonggu
Inbred line	A146	A2M	Lvdahonggu
Inbred line	A147	340D	Lvdahonggu
Inbred line	A150	405	Lvdahonggu
Inbred line	A151	T75	Lvdahonggu
Inbred line	A152	302M	Lvdahonggu
Inbred line	A153	Y09	Lvdahonggu
Inbred line	A168	KY16M	Other
Inbred line	A169	A489	Other
Inbred line	A170	DK516M	Other
Inbred line	A173	4A	Tang SPT
Inbred line	A174	Wu314	Tang SPT
Inbred line	A176	Huangzaosis	Tang SPT
Inbred line	A177	A19 (13N-333)	Tang SPT
Inbred line	A178	S4·4A·ZG	Tang SPT
Inbred line	A179	CJF	Tang SPT
Inbred line	A180	Y85·C72	Tang SPT
Inbred line	A183	926F	Tang SPT
Inbred line	A184	Weike02F	Tang SPT

Inbred line	A187	Yangguang98F	Tang SPT
Inbred line	A189	Dayu3F	Tang SPT
Inbred line	A190	YDF	Tang SPT
Inbred line	A193	S4·4A	Tang SPT
Inbred line	A194	S4	Tropic
Inbred line	A195	S2	Tropic
Inbred line	A196	S5	Tropic
Inbred line	A198	S7	Tropic
Inbred line	A199	Pob501	Tropic
Inbred line	A202	QCY7	unknow
Inbred line	A203	FG-1	unknow
Inbred line	A204	BL-1	unknow
Inbred line	A205	HNHZ4	unknow
Inbred line	A206	YBK-1	unknow
Inbred line	A207	BJN2000M	unknow
Inbred line	A208	TN9F	unknow
Inbred line	A211	Dan340d	Lvdahonggu
Inbred line	A212	F3564	unknow
Inbred line	A215	Liao112	unknow
Inbred line	A216	L118	unknow
Inbred line	A217	L48	unknow
Inbred line	A220	J4112	unknow
Inbred line	A221	Q1261	unknow
Inbred line	A222	Qi205	unknow
Inbred line	A223	U8112	Reid
Inbred line	A225	CAV886	unknow
Inbred line	A232	CIMBL139	Mixed
Inbred line	A235	TN9M	Mixed
Inbred line	A237	DH1M	unknow
Inbred line	A239	H13	Mixed
Inbred line	A240	Weihexin	Mixed
Inbred line	A241	Ao89E4	Mixed
Inbred line	A242	Zun90110	Mixed
Inbred line	A243	H18	Mixed
Inbred line	A244	5003	Mixed
Inbred line	A245	Ye8112	Mixed
Inbred line	A246	Qi318s	Mixed
Inbred line	A247	H23	Mixed
Inbred line	A248	HR962	Mixed
Inbred line	A250	BLY-2	Mixed
Inbred line	A252	H28	Mixed
Inbred line	A253	M3	Mixed
Inbred line	A254	Dibai	Mixed
Inbred line	A255	WAYA24	Mixed
Inbred line	A256	Jiu03	Mixed
Inbred line	A257	H32	Mixed
Inbred line	A258	Ao20	Mixed
Inbred line	A259	Zhonger02	Mixed
Inbred line	A260	Whx89	Mixed
Inbred line	A261	H36	Mixed
Inbred line	A262	Y8H	Mixed
Inbred line	A264	K36	Mixed
Inbred line	A265	H41	Mixed

Inbred line	A266	H45	Mixed
Inbred line	A268	Qun1-1	Mixed
Inbred line	A269	ZH0853	Mixed
Inbred line	A270	H34	Mixed
Inbred line	A271	BNY2	Mixed
Inbred line	A272	K910G	Mixed
Inbred line	A274	H26	Mixed
Inbred line	A275	Qun31	Mixed
Inbred line	A276	DH11F	Mixed
Inbred line	A277	Y9H	Mixed
Inbred line	A278	Z58	Mixed
Inbred line	A279	H15	Mixed
Inbred line	A280	Zheng58370	Reid
Inbred line	A281	Huang7	Mixed
Inbred line	A282	Huotanghuang17	Mixed
Inbred line	A283	GY6	Mixed
Inbred line	A284	Whx101	Mixed
Inbred line	A285	BLY-3	Mixed
Inbred line	A287	Y10H	Mixed
Inbred line	A288	JN2	Mixed
Inbred line	A289	488	Mixed
Inbred line	A291	H31	Mixed
Inbred line	A292	35199	Mixed
Inbred line	A293	Hua160	Mixed
Inbred line	A294	Zao49	Mixed
Inbred line	A295	OAXA179	Mixed
Inbred line	A296	H27	Mixed
Inbred line	A298	WHX48	Mixed
Inbred line	A299	ZX091	Mixed
Inbred line	A302	Qun4-1	Mixed
Inbred line	A303	Jidan261M	Mixed
Inbred line	A304	H22	Mixed
Inbred line	A305	Longkang1	Mixed
Inbred line	A306	Lu65	Mixed
Inbred line	A309	FR218	Mixed
Inbred line	A311	Ziyu3	Mixed
Inbred line	A314	H24	Mixed
Inbred line	A315	J599-2	Mixed
Inbred line	A316	H35	Mixed
Inbred line	A319	H25	Mixed
Inbred line	A320	HN2	Mixed
Inbred line	A322	HN785	Mixed
Inbred line	A325	Han102	Mixed
Inbred line	A326	Yan103	Mixed
Inbred line	A327	Yan172	Mixed
Inbred line	A328	H437	Mixed
Inbred line	A330	Hun21	Mixed
Inbred line	A331	Wu96	Mixed
Inbred line	A334	BEM	unknow
Inbred line	A335	935	Mixed
Inbred line	A336	L01067	unknow
Inbred line	A337	T878	unknow
Inbred line	A338	T877	unknow

Inbred line	A341	Hai13	Mixed
Inbred line	A344	Hai18	Mixed
Inbred line	A345	Hai19	Mixed
Inbred line	A349	Hai23	Mixed
Inbred line	A350	Hai24	Mixed
Inbred line	A351	Hai25	Mixed
Inbred line	A352	Hai26	Mixed
Inbred line	A354	Hai29	Mixed
Inbred line	A356	TJH5F	Mixed
Inbred line	A357	CY189	Mixed
Inbred line	A358	CXY	Tang SPT
Inbred line	A360	XY335X	Mixed
Inbred line	A362	Q1220	Mixed
Inbred line	A363	2013-H-12-0	Mixed
Inbred line	A365	TZDK36	Mixed
Inbred line	A366	QBS1	Mixed
Inbred line	A368	QFC521	Mixed
Inbred line	A369	QKW456-2	Mixed
Inbred line	A370	YF-1	Mixed
Inbred line	A371	JD-1	Mixed
Inbred line	A373	F8-1-11	Mixed
Inbred line	A375	M3564	Mixed
Inbred line	A376	AEF	Mixed
Inbred line	A377	KN-M	Mixed
Inbred line	A378	DG7-1	Mixed
Inbred line	A379	LY-1	Mixed
Inbred line	A380	BJN2000F	Mixed
Inbred line	A381	ZCBN-1	Mixed
Inbred line	A382	BN1029	Mixed
Inbred line	A383	BJ-1	Lancaster
Inbred line	A384	LY16F	unknow
Inbred line	A385	BN189	unknow
Inbred line	A386	BN386	unknow
Inbred line	A387	BN63	unknow
Inbred line	A389	PH6WC	unknow
Inbred line	A390	S37	unknow
Inbred line	A392	Dan340	Lvdahonggu
Inbred line	A393	HuangC	Tang SPT
Inbred line	A395	Zong31	Lvdahonggu
Inbred line	A398	PH4CV	unknow
Inbred line	A399	DK516F	unknow
Inbred line	A402	40M	unknow
Inbred line	A403	40F	unknow
Inbred line	A404	41F	unknow
Inbred line	A405	MY8M	unknow
Inbred line	A406	MY8F	unknow
Inbred line	A407	S35M	unknow
Inbred line	A408	S35F	unknow
Inbred line	A410	12HTJ	unknow
Inbred line	A411	DBM	unknow
Inbred line	A412	QLM	unknow
Inbred line	A413	QLF	unknow
Inbred line	A415	Y22M	unknow

Inbred line	A416	KM	unknow
Inbred line	A417	S36F	unknow
Inbred line	A418	HNFZQB	unknow
Inbred line	A420	12HL-1	unknow
Inbred line	A421	12HT274	unknow
Inbred line	A422	12HT501	unknow
Inbred line	A423	Limin33M	unknow
Inbred line	A424	DH605M	unknow
Inbred line	A425	DH605F	unknow
Inbred line	A426	Dayu3M	unknow
Inbred line	A427	Dayu3Fs	unknow
Inbred line	A428	702M	unknow
Inbred line	A429	DH863F	unknow
Inbred line	A430	13HLD34	unknow
Inbred line	A431	Qi31922G	unknow
Inbred line	A432	XD20M	unknow
Inbred line	A433	XD21M	unknow
Inbred line	A434	XD22M	unknow
Inbred line	A435	10CQ01	unknow
Inbred line	A436	10CQ02	unknow
Inbred line	A439	XD26F	unknow
Landrace	L001	Nong1	Mexico
Landrace	L002	Nong2	Mexico
Landrace	L003	BQYM1050	Mexico
Landrace	L005	BQYM1051	Mexico
Landrace	L007	SSHEB	Mexico
Landrace	L010	XHYM	Mexico
Landrace	L011	CYT	Mexico
Landrace	L014	FENG261	Mexico
Landrace	L015	MYHEB	Mexico
Landrace	L016	CDHYM	Mexico
Landrace	L019	YJB	Mexico
Landrace	L020	HEB	Mexico
Landrace	L022	BQ1094	Mexico
Landrace	L023	BG1106	Mexico
Landrace	L024	XYM1181	Mexico
Landrace	L026	SSHQ1070	Mexico
Landrace	L027	BBG1124	Mexico
Landrace	L028	BH1033	Mexico
Landrace	L030	HYM1042	Mexico
Landrace	L033	BQYM	Mexico
Landrace	L035	XBBG	Mexico
Landrace	L036	HQ1069	Mexico
Landrace	L037	HYM1096	Mexico
Landrace	L038	EFZ1148	Mexico
Landrace	L041	ZSY1177	Mexico
Landrace	L042	HEB1072	Mexico
Landrace	L044	BDH1147	Mexico
Landrace	L045	HQ1093	Mexico
Landrace	L048	HYM1006	Mexico
Landrace	L049	LBYM1149	Mexico
Landrace	L051	HBG	Mexico
Landrace	L053	BYM1131	Mexico

Landrace	L054	EYXHJ	Mexico
Landrace	L059	YJB1088	Mexico
Landrace	L060	BQSZ	Mexico
Landrace	L063	BYQ	Mexico
Landrace	L066	WYBMY	Mexico
Landrace	L068	DBBEB	Mexico
Landrace	L070	BQ1025	Mexico
Landrace	L072	HMY	Mexico
Landrace	L073	YLZ	Mexico
Landrace	L075	BMY	Mexico
Landrace	L076	JHH	Mexico
Landrace	L077	HBG	Mexico
Landrace	L078	XJH	Mexico
Landrace	L080	XJH	Mexico
Landrace	L094	GZH	Mexico
Landrace	L095	BRH	Mexico
Landrace	L096	PWHEB	Mexico
Landrace	L097	QYM	Mexico
Landrace	L098	BEB	Mexico
Landrace	L099	NJZ1004	Mexico
Landrace	L100	XHJ	Mexico
Landrace	L102	LHJ	Mexico
Landrace	L106	106	Mexico
Landrace	L108	108	Mexico
Landrace	L109	109	Mexico
Landrace	L110	110	Mexico
Landrace	L111	111	Mexico
Landrace	L113	113	Mexico
Landrace	L114	114	Mexico
Landrace	L115	115	Mexico
Landrace	L117	117	Mexico
Landrace	L119	119	Mexico
Landrace	L121	121	Mexico
Landrace	L124	124	Mexico
Landrace	L127	127	Mexico
Landrace	L132	132	Mexico
Teosintes	T001	28620	Mexico
Teosintes	T002	Guerrero	Mexico
Teosintes	T003	625	Mexico
Teosintes	T004	No. 14438	Mexico
Teosintes	T005	27215	Mexico
Teosintes	T006	No. 13	Mexico
Teosintes	T007	CIMMYT ID: 29766	Mexico
Teosintes	T008	CIMMYT ID: 29798	Mexico
Teosintes	T009	FS1834	Mexico
Teosintes	T010	FS1841	Mexico
Teosintes	T011	FS1845	Mexico
Teosintes	T012	2460	Mexico
Teosintes	T013	28629	Mexico
Teosintes	T014	III.B. 10	Mexico
Teosintes	T015	Chalco	Mexico
Teosintes	T016	I.A. 7	Mexico
Teosintes	T017	I.B. 29	Mexico

Teosintes	T018	BENZ 967	Mexico
Teosintes	T019	Wilkes 47259	Mexico
Teosintes	T020	FS1818	Mexico
Teosintes	T021	FS1847	Mexico
Teosintes	T022	FS1854	Mexico
Teosintes	T023	IA1	Mexico
Teosintes	T024	IA2	Mexico
Teosintes	T025	IA8	Mexico
Teosintes	T026	IA9	Mexico
Teosintes	T027	IA10	Mexico
Teosintes	T028	IA11	Mexico
Teosintes	T029	IA29	Mexico
Teosintes	T030	IA36	Mexico
Teosintes	T031	BENZ 967	Mexico
Teosintes	T032	Maiz de Pajaro	Mexico
